# Supplementary material for: The association between all-cause mortality and HIV acquisition risk groups in the United States, 2001–2014
Source: PLoS One. 2023 Aug 17;18(8):e0290113. doi: 10.1371/journal.pone.0290113 (PMC10434931; doi:10.1371/journal.pone.0290113)
Supplement: S1 Appendix — (DOCX) [file pone.0290113.s001.docx]

# S1 Appendix. Supplemental Methods

**Study population**

To define the study population, we first identified the population eligible for the mortality analysis (people 18 years or older with linked mortality data), who were administered the questionnaires that allow classification into one of the HIV acquisition risk groups, and had no missing data. Thus, we excluded participants who were not given the necessary questionnaires (N=12,431), or who did not provide answers to the necessary questionnaires (N=5,748). Therefore, 23,657 participants were eligible to be assessed for HIV diagnosis. Then, we restricted to participants without an HIV diagnosis, using HIV antibody test information from NHANES laboratory data and the underlying cause of death from the mortality files (if death occurred). We excluded individuals with a reactive HIV antibody test result (N = 101). We also excluded individuals who did not have a documented, non-reactive HIV antibody test result if their cause of death was “other,” which could include HIV-associated causes of death (N = 152). We also excluded women who had sex with women, unless they had a history of injection drug use (N=1,008). Therefore, our analytic sample included 22,396 participants (Supplemental Figure 1).

**People who ever injected drugs (ever-PWID)**

Because the drug use questionnaires changed among cycles, we used different questions to classify the participants depending on the survey’s cycle. From 2001 to 2004, we created an ever-PWID variable based on the following questions: “1) Have you ever used cocaine or other street drugs? 2) Have you ever used a needle to take street drugs?” From 2005 to 2014, we created the ever-PWID variable based on the following questions: “1) Have you ever used cocaine/heroin/methamphetamine? 2) Have you ever used a needle to inject illegal drugs?”. Only those who reported ever using a needle were considered as ever-PWID.

**Men who have sex with men (MSM)**

We created male sexual orientation variables based on NHANES questions (available from 2001 to 2014): “1) Do you think of yourself as heterosexual or straight, homosexual or gay, bisexual, other? 2) In your lifetime, with how many men have you had sex? 3) In the past 12 months, with how many men have you had sex?” We also included responses to the following NHANES questions (available from 2009 to 2014): “4) In your lifetime, with how many men have you had anal sex? 5) Have you ever had any kind of sex with a man, including oral or anal?”

**Women who have sex with women (WSW)**

We created female sexual orientation variables based on NHANES questions (available from 2001 to 2014): “1) Do you think of yourself as heterosexual or straight, homosexual or lesbian, bisexual, other? 2) In your lifetime, with how many women have you had sex? 3) In the past 12 months, with how many women have you had sex?” We also included responses to the following NHANES questions (available from 2009 to 2014): “4) Have you ever had any kind of sex with a woman? By sex, we mean sexual contact with another woman's vagina or genitals.” We used the WSW variable as an exclusion criterion.

**Covariates of Interest**

**Age-at-risk**

To estimate mortality by age-at-risk, we conducted Lexis expansion to stratify follow-up by intervals of one year, converting the one observation per person to one observation for each time interval (one year of age) per person so that each participant contributes to each age stratum until death or censoring [1]. We further classified individuals into three age groups using tertile cutoff values.

**Education**

We classified participants into three categories: less than high school (i.e., participants who reported “Never attended,” “1^st^-12^th^ Grade,” or “No Diploma”); high school graduate (i.e., responses included “High School Graduate” or “GED or Equivalent”); and more than high school (i.e., participants who reported some college or graduate school).

**Income**

We classified participants into four groups based on self-reported annual family income: 1) $0-14,999, 2) $15,000–34,999, 3) $35,000-64,999, and 4) $65,000 or more.

**Health insurance**

To categorize health insurance status, we used the participants’ answers to the question: “Are you covered by health insurance?”

**Health status**

We classified health status based on self-reported answers to the question: “Would you say your health, in general, is excellent, very good, good, etc.?” Participants who answered “Excellent,” “Very good,” or “Good” were considered to be in “good health,” and participants who answered “Fair” and “Poor” were considered to be in “fair health” and “poor health,” respectively.

**Alcohol and tobacco use**

Per the National Institute on Alcohol Abuse and Alcoholism (NIAAA) guidelines, binge drinking is defined as the consumption of five drinks for men or four drinks for women within two hours [2]. Therefore, we defined “at-risk alcohol use” as 12 or more days per year in which a participant had at least five (men) or four (women) drinks of any alcoholic beverage. We classified participants into four groups: 1) no alcohol consumption within the past year, 2) had alcohol consumption but never had binge drinking episodes within the past year, 3) less than 12 binge drinking episodes, and 4) more than 12 binge drinking episodes [3, 4]. “Smokers” were defined in NHANES as participants who smoked at least 100 cigarettes over their lifetime [5].

**Body Mass Index**

We defined body mass index (BMI) as a participant's weight in kilograms divided by the square of height in meters and obesity as BMI above 30 kg/m^2^ according to the World Health Organization definition [6].

**Condom use**

We created a “condom use” variable based on whether the participant reported sexual activity and condom use in the past year. We classified participants who reported having had sex without a condom at least once in the last 12 months as “didn’t use condoms in the past year.” We classified participants who reported never having had sex without using a condom in the last 12 months as “used condoms in the past year.”

**Lifetime sexual partners**

We classified individuals as “without multiple sexual partners,” if they reported having no or one sexual partner in their lifetime. If they had more than one partner in their lifetime, they were classified as “multi-partner.”

**History of sexually transmitted infections**

We created a sexually transmitted infection (STI) variable based on the self-reported question: “Has a doctor or other health care professional ever told you that you had genital herpes, genital warts, or gonorrhea?” If the participant answered “Yes” to any of the questions, they were considered to have a history of an STI. Otherwise, they were considered to have no known STI history.

**Proportional hazard assumption**

Additionally, we assessed the Cox proportional hazard assumption using two standard approaches: 1) to visually assess the parallelisms of the lines of the graph of the log of -log survival versus the log of survival time (log-negative log plot), and 2) to include and assess the significance of the time-dependent variable in the model. If the lines were parallel and the time-dependent variable was not significant, then we considered the proportional hazard assumption to not be violated.

**Sensitivity analysis**

We also performed a sensitivity analysis, in which we included participants in the adjusted Cox proportional hazards model who had been previously excluded due to the inability to classify them into HIV acquisition risk groups because of missing data or not being asked questions necessary for categorization.

**References**

1. Nitika, Mishra SS, Lohani P. Lexis expansion: a prerequisite for analyzing time changing variables in a cohort study. Nepal J Epidemiol. 2017;7(2):681-4. doi: 10.3126/nje.v7i2.17974. PubMed PMID: 29181229.

2. National Institute on Alcohol Abuse and Alcoholism. Understanding the impact of alcohol on human health and well-being 2021 [cited 2022 October 31]. Available from: Available at: <https://www.niaaa.nih.gov/alcohol-health/overview-alcohol-consumption/moderate-binge-drinking>.

3. Piano MR, Burke L, Kang M, Phillips SA. Effects of repeated binge drinking on blood pressure levels and other cardiovascular health metrics in young adults: National Health and Nutrition Examination Survey, 2011-2014. J Am Heart Assoc. 2018;7(13). doi: 10.1161/JAHA.118.008733. PubMed PMID: 29950486.

4. Cochran SD, Mays VM. Sexual orientation and mortality among US men aged 17 to 59 years: results from the National Health and Nutrition Examination Survey III. Am J Public Health. 2011;101(6):1133-8. doi: 10.2105/AJPH.2010.300013. PubMed PMID: 21493941.

5. Centers for Disease Control and Prevention (CDC). National Center for Health Statistics (NCHS). National Health and Nutrition Examination Survey sample design, survey questionnaire, examination protocol, laboratory protocol, survey data, and analytic guidelines [2001-2014] [cited 2022 October 31]. Available from: Available at: <https://www.cdc.gov/nchs/nhanes/index.htm>.

6. World Health Organization. Obesity 2022 [cited 2022 October 31]. Available from: Available at: <https://www.who.int/health-topics/obesity#tab=tab_1>.
