# Supplementary material for: The association between all-cause mortality and HIV acquisition risk groups in the United States, 2001–2014
Source: PLoS One. 2023 Aug 17;18(8):e0290113. doi: 10.1371/journal.pone.0290113 (PMC10434931; doi:10.1371/journal.pone.0290113)
Supplement: S2 Appendix — (DOCX) [file pone.0290113.s002.docx]

# S2 Appendix. Supplemental Results

*Associations of population characteristics with HIV acquisition risk groups*

Significant associations were evident between risk groups and age, gender, education, health status, smoking status, condom use, alcohol use, and history of STI (Figure 1, Supplemental Table 1). Therefore, we assessed these variables as potential confounders of the relationship between risk group and mortality.

*Associations of mortality with population characteristics*

The unadjusted associations of all-cause mortality rate with population characteristics are presented in Supplemental Table 2. Among all risk groups together, the weighted all-cause mortality rate among males was higher than among females (493.4/100,000 PY vs. 314.3/100,000 PY). Higher mortality rates were observed among participants who were: Black and Hispanic (compared with non-Hispanic White); with PIR < 1 (compared with PIR > 1); with less than high school education (compared with high school or more); with annual family income <$15,000 (compared with >$15,000); in poor or fair health (compared with good health); with at-risk alcohol consumption (compared with fewer than 12 binge drinking episodes in the past year); with tobacco smoking (compared with <100 lifetime cigarettes); obese (compared with non-obese); with >11 lifetime sexual partners (compared with <2 lifetime sexual partners).

*Mortality association with not mutually exclusive HIV acquisition risk groups*

Because some participants met the criteria for more than one HIV acquisition risk group (e.g., MSM who have ever used injection drugs), therefore, in this sensitivity analysis, we categorized participants into multiple HIV acquisition risk groups (e.g., an MSM who injects drugs contributes to both ever-PWID and MSM). (Supplemental Table 4) The highest weighted all-cause mortality rate was observed among ever-PWID (1166.4 deaths/100,000 PY), followed by MSM (650.0 deaths/100,000 PY) and HIH (617.7 deaths/100,000 PY). We observed the lowest weighted all-cause mortality rate among those who are not HIH (361.6 deaths/100,000 PY).
