# Supplementary material for: The association between all-cause mortality and HIV acquisition risk groups in the United States, 2001–2014
Source: PLoS One. 2023 Aug 17;18(8):e0290113. doi: 10.1371/journal.pone.0290113 (PMC10434931; doi:10.1371/journal.pone.0290113)
Supplement: S3 Appendix — (DOCX) [file pone.0290113.s003.docx]

# S3 Appendix. Supplemental Tables

| **Covariate** | **HIV Acquisition Risk Group, N (%) ^a^** | | | | | |
| --- | --- | --- | --- | --- | --- | --- |
|  | **Ever-PWID**  **N=509** | **MSM**  **N=570** | **HIH**  **N=4,498** | **HAH**  **N=16,820** | **p-value ^b, c^** |  |
| **Age at risk, tertiles ^d^** |  |  |  |  | < 0.0001 |  |
| 18 – < 44 years | 99 (1.77) | 188 (2.79) | 2,120 (21.14) | 5,222 (74.29) |  |  |
| > 44 – <59 years | 202 (2.59) | 199 (2.86) | 1,406 (11.96) | 5,809 (82.59) |  |  |
| > 59 years | 208 (2.37) | 178 (2.6) | 1,032 (7.83) | 5,733 (87.2) |  |  |
| **Gender** |  |  |  |  | < 0.0001 |  |
| Male | 340 (2.89) | 565 (5.27) | 2,133 (12.15) | 8,488 (79.69) |  |  |
| Female | 169 (1.57) |  | 2,425 (14.88) | 8,276 (83.55) |  |  |
| **Race/ Ethnicity** |  |  |  |  | < 0.0001 |  |
| Hispanic | 76 (1.21) | 143 (2.44) | 1,645 (27.92) | 3,950 (68.43) |  |  |
| Non-Hispanic White | 301 (2.53) | 292 (3) | 1,499 (9.07) | 7,912 (85.4) |  |  |
| Black/African American | 105 (2) | 94 (1.86) | 1,116 (23.27) | 3,474 (72.86) |  |  |
| Other | 27 (2.06) | 36 (2.28) | 298 (12.59) | 1,428 (83.06) |  |  |

**Supplemental Table 1.** Distribution of demographic characteristics stratified by mutually exclusive HIV acquisition risk group among participants in 2001-2014 NHANES

**Supplemental Table 1 continued.** Distribution of demographic characteristics stratified by mutually exclusive HIV acquisition risk group among participants in 2001-2014 NHANES

| **Covariate** | **Risk Group, N (%) ^a^** | | | | |
| --- | --- | --- | --- | --- | --- |
|  | **Ever-PWID** | **MSM** | **HIH** | **HAH** | **p-value ^b, c^** |
| **Education** |  |  |  |  | < 0.0001 |
| Less than high school | 135 (3.44) | 78 (1.45) | 1,934 (32.94) | 2,918 (62.18) |  |
| High school | 167 (3.38) | 113 (2.16) | 1,181 (15.73) | 3,758 (78.74) |  |
| More than high school | 207 (1.56) | 374 (3.29) | 1,442 (7.87) | 10,081 (87.28) |  |
| **Annual Family Income, $** |  |  |  |  | < 0.0001 |
| < 15,000 | 152 (4.65) | 107 (3.24) | 2,951 (76.76) | 520 (15.35) |  |
| 15,000 – 34,999 | 172 (3.25) | 133 (2.39) | 1,535 (18.85) | 4,306 (75.5) |  |
| 35,000 – 64,999 | 88 (1.74) | 150 (3.17) | 72 (0.76) | 5,161 (94.34) |  |
| ≥ 65,000 | 72 (1.1) | 152 (2.33) | 0 (0.0) | 6,777 (96.56) |  |

**Supplemental Table 1 continued.** Distribution of demographic characteristics stratified by mutually exclusive HIV acquisition risk **group among participants in 2001-2014 NHANES**

| **Covariate** | **Risk Group, N (%) ^a^** | | | | |
| --- | --- | --- | --- | --- | --- |
|  | **Ever-PWID** | **MSM** | **HIH** | **HAH** | **p-value ^b, c^** |
| **Health Insurance** |  |  |  |  | < 0.0001 |
| No | 197 (4.35) | 147 (2.7) | 2,036 (28.18) | 3,551 (64.76) |  |
| Yes | 308 (1.68) | 417 (2.76) | 2,516 (9.6) | 13,201 (85.96) |  |
| **Health Status** |  |  |  |  |  |
| Good |  |  |  |  |  |
| Fair |  |  |  |  |  |
| Poor |  |  |  |  |  |
| **At-risk Alcohol Consumption** |  |  |  |  | < 0.0001 |
| No drinking in the past year | 142 (3.85) | 86 (2.53) | 811 (16.18) | 2,350 (77.44) |  |
| No binge drinking episodes/year | 141 (1.56) | 223 (2.68) | 1,479 (10.79) | 6,972 (84.97) |  |
| < 12 binge drinking episodes/year | 66 (1.87) | 95 (3.06) | 546 (10.62) | 2,577 (84.45) |  |
| ≥12 binge drinking episodes/year | 153 (4.21) | 129 (3.84) | 840 (14.76) | 2,725 (77.19) |  |
| **Smoking Status** |  |  |  |  |  |
| < 100 lifetime cigarettes | 52 (0.45) | 247 (2.26) | 2,217 (11.86) | 9,684 (85.44) | < 0.0001 |
| > 100 lifetime cigarettes | 456 (4.51) | 309 (3.36) | 2,159 (14.87) | 6,809 (77.26) |  |

**Supplemental Table 1 continued.** Distribution of demographic characteristics stratified by mutually exclusive HIV transmission risk

| **Covariate** | **Risk Group, N (%) ^a^** | | | | | | | | | |
| --- | --- | --- | --- | --- | --- | --- | --- | --- | --- | --- |
|  | **Ever-PWID** | | **MSM** | | | **HIH** | **HAH** | | **p-value ^b, c^** | |
| **Body Mass Index (kg/m^2^)** | |  | |  |  | | |  | | .002 |
| BMI < 30 kg/m^2^ | 358 (2.52) | | 391 (2.92) | | | 2,835 (12.97) | 10,684 (81.59) | | |  |
| BMI > 30 kg/m^2^ | 150 (1.81) | | 169 (2.43) | | | 1,665 (14.18) | 5,961 (81.58) | | |  |
| **Condom Use** ^e^ |  | |  | | |  |  | | 0.026 | |
| No | 252 (2.52) | | 276 (2.67) | | | 2,351 (14.17) | 8,068 (80.64) | |  | |
| Yes | 69 (2.43) | | 87 (3.39) | | | 734 (16.23) | 2,191 (77.94) | |  | |
| **Lifetime Sexual Partners** ^e^**, n** |  | |  | | |  |  | | < 0.0001 | |
| < 2 | 19 (0.44) | | 37 (0.65) | | | 1,078 (15.2) | 3,593 (83.7) | |  | |
| 2 – 11 | 123 (0.87) | | 203 (1.81) | | | 2,437 (13.44) | 9,190 (83.88) | |  | |
| ≥ 11 | 363 (6.55) | | 323 (6.34) | | | 1,028 (12.05) | 3,960 (75.06) | |  | |
| **History of STI** ^e^ |  | |  | | |  |  | | < 0.0001 | |
| No | 389 (2.19) | | 455 (2.72) | | | 3,964 (14.33) | 13,830 (80.77) | |  | |
| Yes | 62 (4.25) | | 37 (2.42) | | | 210 (9.62) | 1,046 (83.71) | |  | |

group among participants in 2001-2014 NHANES**NHANES**, National Health, and Nutrition Examination Survey; **MSM**, men who have sex with men; **PWID**, people who inject drugs; **HIH**, heterosexually active people at increased risk for HIV; **HAH**, heterosexually active people at average risk for HIV; **PIR**, poverty-income ratio; **BMI**, body mass index; **SES**, socioeconomic status; **STI**, sexually transmitted infection

^a^ Row weighted percentage.

^b^ Adjusted for the design weights, strata, and primary sampling units.

^c^ Rao-Scott F adjusted chi-square test.

^d^ The time of death or the time of exit from the study.

^e^ Among participants who ever had sex.

**Supplemental Table 2.** All-cause mortality rates by population characteristics

| **Covariate** | **Participants,**  **N (%)^a^** | **Deaths,**  **N** | **Length of follow-up, years** | **Unweighted all-cause mortality rate per 100,000 PY^b^** | **Weighted all-cause mortality rate per 100,000 PY^c^** | **p-value ^d,e^** |
| --- | --- | --- | --- | --- | --- | --- |
| **Overall** | 22,396 (100) | 1,139 | 249,326 | 456.83 | 407.15 |  |
| **Age at risk, tertiles** ^e^ |  |  |  |  |  | <0.0001 |
| 18 – < 44 years | 7,888 (33.6) | 101 | 24,513 | 412.0 | 348.6 |  |
| > 44 – < 59 years | 7,871 (34.1) | 237 | 74,953 | 316.2 | 271.9 |  |
| > 59 years | 6,638 (32.3) | 336 | 63,414 | 529.9 | 412.1 |  |
| **Gender** |  |  |  |  |  | <0.0001 |
| Male | 11,526 (52.23) | 725 | 126,618 | 572.59 | 493.44 |  |
| Female | 10,870 (47.77) | 414 | 122,708 | 337.39 | 314.33 |  |
| **Race/Ethnicity** |  |  |  |  |  | <0.0001 |
| Hispanic | 5,814 (13.8) | 191 | 65,556 | 291.36 | 253.57 |  |
| Non-Hispanic White | 10,004 (69.19) | 576 | 115,070 | 500.56 | 418.85 |  |
| Black/African American | 4,789 (11.05) | 331 | 52,345 | 632.35 | 577.17 |  |
| Other ^f^ | 1,789 (5.96) | 41 | 16,356 | 250.68 | 299.75 |  |

**Supplemental Table 2 continued.** All-cause mortality rates by population characteristics

| **Covariate** | **Participants,**  **N (%)^a^** | **Deaths,**  **N** | **Length of follow-up, years** | **Unweighted all-cause mortality rate per 100,000 PY^b^** | | **Weighted all-cause mortality rate per 100,000 PY^c^** | | **p-value^d,e^** | |
| --- | --- | --- | --- | --- | --- | --- | --- | --- | --- |
| **Education**^f^ |  |  |  |  | |  | | <0.0001 | |
| Less than high school | 5,065 (15.07) | 352 | 56,682 | 621.01 | | 658.81 | |  | |
| High school | 5,219 (23.03) | 325 | 58,271 | 557.74 | | 509.62 | |  | |
| More than high school | 12,104 (61.86) | 461 | 134,288 | 343.29 | | 307.11 | |  | |
| **Annual Family Income, $**^f^ | |  |  | |  | |  | <0.0001 | |
| < 15,000 | 3,730 (11.99) | 329 | 41,127 | | 799.96 | | 757.47 |  | |
| 15,000 – 34,999 | 6,146 (21.51) | 361 | 68,444 | | 527.44 | | 497.94 |  | |
| 35,000 – 64,999 | 5,471 (25.63) | 244 | 62,133 | | 392.71 | | 386.56 |  | |
| ≥ 65,000 | 7,001 (40.67) | 198 | 77,104 | | 256.80 | | 262.30 |  | |
| **Socioeconomic Status** |  |  |  | |  | |  | <0.0001 | |
| Above the federal poverty level “PIR ≥ 1” | 17,533 (85.62) | 796 | 197,891 | | 402.24 | | 367.92 |  | |
| At or below the federal poverty level “PIR < 1” | 4,815 (14.38) | 336 | 50,917 | | 659.90 | | 643.22 |  | |
| **Health Insurance**^f^ |  |  |  |  | |  | | 0.343 | |
| No | 5,931 (20.71) | 284 | 66,407 | 427.66 | | 432.50 | |  | |
| Yes | 16,442 (79.29) | 854 | 182,640 | 467.59 | | 400.18 | |  | |
| **Health Status**^f^ |  |  |  |  | |  | | <0.0001 | |
| Good | 17,951 (85.08) | 651 | 202,906 | 320.84 | | 304.14 | |  | |
| Fair | 3,775 (12.68) | 363 | 39,797 | 912.12 | | 893.20 | |  | |
| Poor | 652 (2.16) | 125 | 6,427 | 1944.86 | | 1978.55 | |  | |
|  |  |  |  |  | |  | |  |  |
|  |  |  |  |  | |  | |  |  |

**Supplemental Table 2 continued.** All-cause mortality rates by population characteristics

| **Covariate** | **Participants,**  **N (%)^a^** | **Deaths,**  **N** | | **Length of follow-up, years** | | **Unweighted all-cause mortality rate per 100,000 PY^b^** | | **Weighted all-cause mortality rate per 100,000 PY^c^** | | **p-value ^d,e^** | |
| --- | --- | --- | --- | --- | --- | --- | --- | --- | --- | --- | --- |
| **At-risk Alcohol Consumption**^f^ |  |  | |  | |  | |  | | <0.0001 | |
| No drinking in the past year | 3,389 (13.52) | 329 | | 37,513 | | 877.02 | | 839.50 | |  | |
| No binge drinking episodes/year | 8,815 (40.66) | 358 | | 99,185 | | 360.94 | | 325.30 | |  | |
| < 12 binge drinking episodes/year | 3,284 (17.03) | 124 | | 36,811 | | 336.85 | | 310.05 | |  | |
| ≥12 binge drinking episodes/year | 3,847 (18.17) | 229 | | 42,774 | | 535.37 | | 441.50 | |  | |
| **Smoking Status** ^f^ |  |  | |  | |  | |  | | <0.0001 | |
| <100 lifetime cigarettes | 12,200 (54.75) | 337 | | 136,255 | | 247.33 | | 218.16 | |  | |
| >100 lifetime cigarettes | 9,733 (45.25) | 798 | | 108,855 | | 733.08 | | 637.95 | |  | |
| **Body Mass Index** ^f^ | | |  | |  | |  | |  | | <0.0001 |
| BMI < 30 kg/m^2^ | 14,268 (65.64) | 652 | | 160,348 | | 406.62 | | 345.61 | |  | |
| BMI > 30 kg/m^2^ | 7,945 (34.36) | 464 | | 86,785 | | 534.66 | | 511.48 | |  | |
| **Condom Use** |  |  | |  | |  | |  | | 0.725 | |
| No | 10,947 (80.74) | 360 | | 112,189 | | 320.89 | | 296.19 | |  | |
| Yes | 3,081 (19.26) | 103 | | 30,510 | | 337.60 | | 311.16 | |  | |
| **Lifetime Sexual Partners** ^f,g^ | | |  | |  | |  | |  | | 0.082 |
| < 2 | 4,727 (19.81) | 191 | | 52,769 | | 361.96 | | 333.51 | |  | |
| 2 – 11 | 11,953 (54.27) | 563 | | 133,707 | | 421.07 | | 368.33 | |  | |
| ≥ 11 | 5,674 (25.78) | 382 | | 62,288 | | 613.28 | | 549.08 | |  | |
| **History of STI** |  |  | |  | |  | |  | | 0.756 | |
| No | 18,638 (92.31) | 832 | | 213,758 | | 389.22 | | 365.52 | |  | |
| Yes | 1,355 (7.69) | 61 | | 15,883 | | 384.06 | | 345.25 | |  | |

**PY,** person-year; **PIR**, poverty-income ratio; **BMI,** body mass index

^a^ Weighted percentage.

^b^ Unweighted, rounded to one full year.

^c^ Adjusted for the design weights, strata, and primary sampling units.

^d^ P-value from Cox proportional hazards regression models.

^e^ The time of death or the time of exit from the study as per the Lexis expansion.

^f^ The remaining percentage is from those with missing data of the row variable.

^g^ Among participants who reported ever having sex.

**Supplemental Table 3.** All-cause mortality rates among participants in 2001-2014 NHANES, by age and mutually exclusive HIV acquisition risk groups^a,b,c^

| **HIV acquisition risk groups ^d^** | **18 - < 44 years** | **>44 – <59 years** | **> 59 years** | **P-value of interaction^e^** |
| --- | --- | --- | --- | --- |
| Male HAH | 262.39 | 389.55 | 634.77 | .091 |
| Female HAH | 101.93 | 288.54 | 326.78 |  |
| Male ever-PWID | 1359.99 | 853.88 | 1224.58 |  |
| Female ever-PWID | 1832.81 | 1878.65 | 342.31 |  |
| MSM | 497.72 | 572.95 | 802.02 |  |
| Male HIH | 229.03 | 930.19 | 1161.94 |  |
| Female HIH | 325.25 | 622.28 | 963.83 |  |

**NHANES**, National Health, and Nutrition Examination Survey; **MSM**, men who have sex with men; **PWID**, people who inject drugs **HIH**, heterosexually active people at increased risk for HIV; **HAH**, heterosexually active people at average risk for HIV

^a^ Weighted all-cause mortality rate per 100,000 person-year.

^b^ The time of death or the time of exit from the study as per the Lexis expansion.

^c^ Age categorized in tertiles.

^d^ Mutually exclusive HIV risk acquisition groups.

^e^ Adjusted for the design weights, strata, and primary sampling units.

**Supplemental Table 4.** All-cause mortality rates by not mutually exclusive HIV acquisition risk groups

| **Covariate** | **Participants,**  **N (%)^a^** | **Deaths,**  **N** | **Length of follow-up, years^b^** | **Unweighted all-cause mortality rate per 100,000 PY** | **Weighted all-cause mortality rate per 100,000 PY^c^** |
| --- | --- | --- | --- | --- | --- |
| **Ever-PWID** ^f,i^ |  |  |  |  |  |
| No | 21,871 (97.74) | 1,063 | 243,713 | 436.17 | 390.31 |
| Yes | 509 (2.26) | 76 | 5,390 | 1409.89 | 1166.42 |
| **MSM**^g, i^ |  |  |  |  |  |
| No | 10,916 (94.44) | 683 | 120,079 | 568.79 | 484.66 |
| Yes | 607 (5.56) | 42 | 6,516 | 644.61 | 650.02 |
| **HIH**^f,i^ | | |  |  |  |
| No | 17,009 (85.6) | 762 | 192,182 | 396.50 | 361.59 |
| Yes | 4,690 (14.4) | 322 | 49,660 | 648.41 | 617.70 |

**PY,** person-year; **MSM**, men who have sex with men; **PWID**, people who inject drugs; **HIH**, heterosexually active people at increased risk for HIV

^a^ Weighted percentage.

^b^ Unweighted, rounded to one full year.

^c^ Adjusted for the design weights, strata, and primary sampling units.

^e^ The time of death or the time of exit of the study as per the Lexis expansion.

^f^ The remaining percentage is from those with missing data of the row variable.

^g^ Among male participants.

^i^ Not mutually exclusive HIV risk acquisition groups.

**Supplemental Table 5**. Unadjusted and adjusted association of mutually exclusive risk groups and mortality in the sample that includes those with unknown risk group category, participants in 2001-2014 NHANES

|  | **Unadjusted ^a,b^** |  | **Adjusted ^a,b,c^** |
| --- | --- | --- | --- |
| **HIV Acquisition Risk Group** | **All-cause mortality HR**  **(95% CI)** |  | **All-cause mortality HR**  **(95% CI)** |
| HAH | Ref. |  | Ref. |
| Ever-PWID | 3.32 (2.48, 4.44) |  | 2.30 (1.71, 3.09) |
| MSM | 1.78 (1.19, 2.67) |  | 1.68 (1.12, 2.52) |
| HIH | 1.68 (1.43, 1.97) |  | 2.10 (1.79, 2.48) |
| Unknown, question not answered | 1.60 (1.34, 1.90) |  | 1.30 (1.06, 1.58) |
| Unknown, question not given | 9.01 (8.23, 9.86) |  | 1.36 (1.18, 1.57) |

**NHANES**, National Health, and Nutrition Examination Survey; **HR**, hazard ratio; **CI**, confidence interval; **MSM**, men who have sex with men; **PWID**, people who inject drugs **HIH**, heterosexually active people at increased risk for HIV; **HAH**, heterosexually active people at average risk for HIV

^a^ Cox proportional hazards regression model

^b^ Adjusted for the design weights, strata, cluster, and primary sampling units

^c^ Adjusted for age, race, education, smoking status, and at-risk alcohol consumption
