# Supplementary material for: The association between all-cause mortality and HIV acquisition risk groups in the United States, 2001–2014
Source: PLoS One. 2023 Aug 17;18(8):e0290113. doi: 10.1371/journal.pone.0290113 (PMC10434931; doi:10.1371/journal.pone.0290113)
Supplement: S4 Appendix — (DOCX) [file pone.0290113.s004.docx]

# S4 Appendix. Supplemental Figures

**FIGURE LEGENDS**

**Supplemental Fig 1. Inclusion criteria**

This figure presents the flow chart of the inclusion and exclusion criteria. We display the unweighted numbers and the weighted percentages. Individuals are classified into four mutually exclusive groups, ever-**PWID**, people who ever injected drugs; **MSM**, men who have sex with men; **HIH**, heterosexually active people at increased risk for HIV; **HAH**, heterosexually active people at average risk for HIV; **WSW**, women who have sex with women. Groups placed in shaded rectangles represent the groups at increased risk of HIV acquisition.

Increased risk for HIV acquisition

**Supplemental Fig 1. Inclusion criteria**
